# Anti-platelet drugs attenuate the expansion of circulating CD14^high^CD16^+^ monocytes under pro-inflammatory conditions

**DOI:** 10.1093/cvr/cvw089

**Published:** 2016-04-26

**Authors:** Kerry Layne, Paolo Di Giosia, Albert Ferro, Gabriella Passacquale

**Affiliations:** Department of Clinical Pharmacology, Cardiovascular Division, British Heart Foundation Centre for Research Excellence, King's College London, Franklin-Wilkins Building, 150 Stamford Street, London SE1 9NH, UK

**Keywords:** Monocytes, Aspirin, Clopidogrel, Platelets, Inflammation

## Abstract

**Aims:**

Levels of circulating CD14^high^CD16^+^ monocytes increase in atherosclerotic patients and are predictive of future cardiovascular events. Platelet activation has been identified as a crucial determinant in the acquisition of a CD16^+^ phenotype by classical CD14^high^CD16^−^ cells. We tested the hypothesis that anti-platelet drugs modulate the phenotype of circulating monocytes.

**Methods and results:**

Sixty healthy subjects undergoing influenza immunization were randomly assigned to either no treatment or anti-platelet therapy, namely aspirin 300 mg or 75 mg daily, or clopidogrel (300 mg loading dose followed by 75 mg), for 48 h post-immunization (*n* = 15/group). Monocyte subsets, high-sensitivity C-reactive protein, pro-inflammatory cytokines, and P-selectin were measured at baseline and post-immunization. The CD14^high^CD16^+^ monocyte cell count rose by 67.3% [interquartile range (IQR): 35.7/169.2; *P* = 0.0002 vs. baseline] in untreated participants. All anti-platelet regimes counteracted expansion of this monocytic subpopulation. Although no statistical differences were noted among the three treatments, aspirin 300 mg was the most efficacious compared with the untreated group (−12.5% change from baseline; IQR: −28.7/18.31; *P* = 0.001 vs. untreated). Similarly, the rise in P-selectin (17%; IQR: −5.0/39.7; *P* = 0.03 vs. baseline) observed in untreated participants was abolished by all treatments, with aspirin 300 mg exerting the strongest effect (−30.7%; IQR: −58.4/−0.03; *P* = 0.007 vs. untreated). Changes in P-selectin levels directly correlated with changes in CD14^high^CD16^+^ cell count (*r* = 0.5; *P* = 0.0002). There was a similar increase among groups in high-sensitivity C-reactive protein (*P* < 0.03 vs. baseline levels).

**Conclusions:**

Anti-platelet drugs exert an immunomodulatory action by counteracting CD14^high^CD16^+^ monocyte increase under pro-inflammatory conditions, with this effect being dependent on the amplitude of P-selectin reduction.

## 1. Introduction

Circulating human monocytes consist of a heterogeneous cell population generally classified into three main subtypes: the ‘classical’ CD14^high^CD16^−^, ‘intermediate’ CD14^high^CD16^+^, and ‘non-classical’ CD14^low^CD16^+^ cells.^1^ The prevalence of these subpopulations in the peripheral blood changes under pro-inflammatory conditions, with an expansion of the ‘intermediate’ subset typically occurring in the context of atherosclerosis-related inflammation.^2^ Although the functional implications of such an event in terms of disease progression remain elusive, a positive correlation between the level of CD14^high^CD16^+^ monocytes and the presence of subclinical atherosclerosis has been demonstrated.^3^ More importantly, intermediate monocytes have proved to be an independent predictive factor for future cardiovascular events,^4^ and good correlation between their blood level and coronary plaque vulnerability has been detected.^5–8^ This indicates that CD14^high^CD16^+^ cells may represent a novel biomarker of cardiovascular risk and a potential target for preventative therapeutic strategies.

In this respect, our previous work suggests that anti-platelet drugs may have a modulatory action on the phenotype of circulating monocytes. Indeed, we have found that platelet activation is a key determinant in the acquisition of a CD16^+^ profile by human monocytes.^9^ Furthermore, using ApoE^−/−^ mice, we have demonstrated the efficacy of platelet inhibition *in vivo*, as achieved by either aspirin or clopidogrel administration, in counteracting the blood monocytosis and expansion of circulating Ly6C^low^ cells, i.e. the murine counterpart of CD16 monocytes that accompany disease progression in this well-established animal model of atherosclerosis.^10^ In the current clinical study, we have assessed the effect of anti-platelet drugs currently used in cardiovascular prophylaxis on the phenotype of circulating monocytes in healthy human subjects. We have used the influenza immunization as an experimental model of acute inflammation, in line with our previous report showing an increase in the pool of circulating CD14^high^CD16^+^ cells in response to vaccine administration,^9^ and also in keeping with other researchers who have previously used immunizations, particularly the seasonal influenza and *Salmonella typhi* immunizations, as stimuli in clinical studies to generate mild, systemic inflammation and investigate cardiovascular physiology.^11–13^

## 2. Methods

### 2.1 Study design

Sixty healthy subjects were studied before and 48 h after receiving the seasonal influenza immunization in agreement with our previously published work.^9^ Subjects were recruited from workers employed by Guy's and St Thomas' National Health Service Foundation Trust (GSTT) who attended the Occupational Health Department requesting influenza immunization. Recruitment criteria were as follows: age >18 years with no significant past medical history and taking no regular prescribed, herbal, or over-the-counter medications. None of the subjects had taken anti-platelet or anti-inflammatory drugs in the preceding fortnight, and the cut-off value for high-sensitivity C-reactive protein at baseline was 2 mg/L. Participants were randomly assigned to receive either anti-platelet therapy or no treatment, according to the following scheme:

Group 1 (aspirin 300, *n* = 15): aspirin 300 mg once daily for 48 h following immunization

Group 2 (aspirin 75, *n* = 15): aspirin 75 mg once daily for 48 h following immunization

Group 3 (clopidogrel, *n* = 15): initial loading dose of clopidogrel 300 mg orally following immunization, with a further dose of clopidogrel 75 mg 24 h later

Group 4 (untreated, *n* = 15): no anti-platelet treatment given following immunization

The first dose of the drug was administrated concomitantly to influenza vaccination, and directly observed tablet taking was carried out by the investigators.

The clinical study was reviewed and given favourable opinion by the NRES London—Dulwich Research Ethics Committee (ref. number 13/LO/1664) and registered on the UK Clinical Research Network Portfolio (South London network study identification number 16644). All participants gave informed consent. The study was performed conforming to the Declaration of Helsinki.

### 2.2 Influenza immunization

The 2014/15 National Health Service trivalent seasonal influenza immunization, an inactivated (split virion) vaccination, specifically targeting ‘A/California/7/2009 (H1N1)pdm09-like virus’, ‘A/Texas/50/2012 (H3N2)-like virus’, and ‘B/Massachusetts/2/2012-like virus’, manufactured by Sanofi Pasteur MSD Limited, was administered via intramuscular injection into the upper arm by a trained healthcare professional.

### 2.3 Blood collection

Venous blood samples were collected immediately prior to immunization and again 48 h later. Samples were processed at ViaPath laboratory at GSTT, London (UK) for measurement of triglycerides, total cholesterol, low-density lipoprotein (LDL) cholesterol, high-density lipoprotein (HDL) cholesterol, and HbA1c at baseline only. Full blood count and high-sensitivity C-reactive protein was measured at baseline and 48 h post-immunization using a Dade Behring BN II automated analyser to perform an assay utilizing polystyrene particles coated with monoclonal antibody and fixed-time kinetic nephelometric measurements.^14^ Additional samples were obtained at both time points for whole blood flow cytometry to characterize monocyte phenotypes (as detailed in the following paragraphs), for serum collection to measure pro-inflammatory cytokines and plasma citrate for P-selectin assessment. Blood sampling at baseline and post-vaccination took place at the same time to exclude circadian variation.

### 2.4 Whole blood flow cytometry analysis

**Figure 1:**
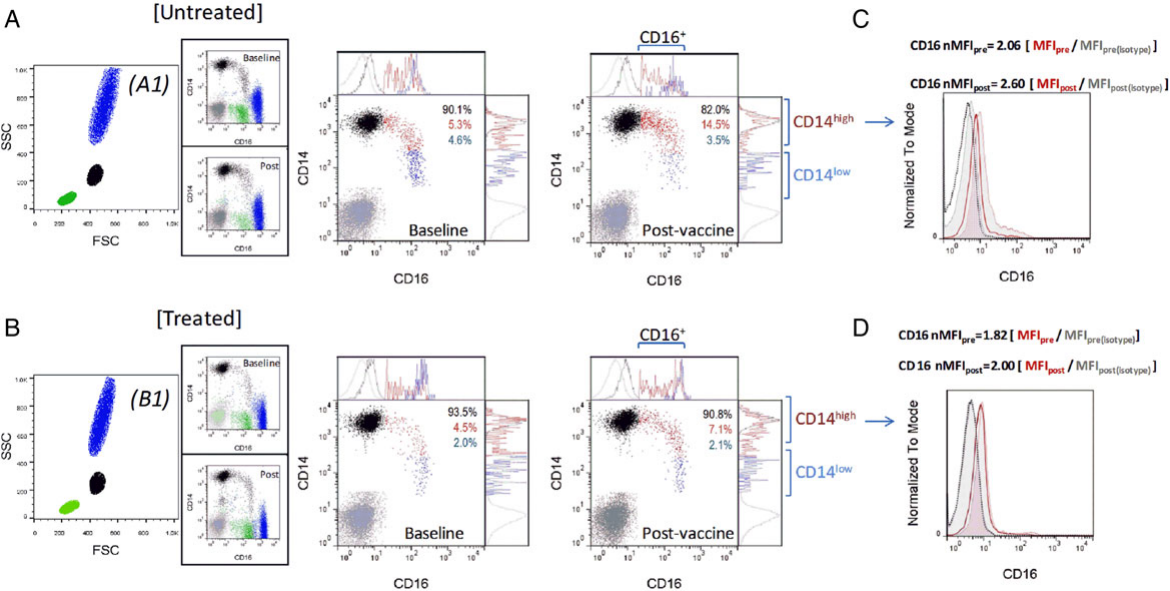
Representative flow cytometry data. Dot plots representative of untreated (*A*) and treated (*B*) participants are shown. Expression of CD14 and CD16 was analysed within the monocyte gated population identified on a FSC vs. SSC plot (*A1* and *B1*, in black), enabling distinction of CD14^high^CD16^−^ (black), CD14^high^CD16^+^ (red), and CD14^low^CD16^+^ (blue) cells (the isotype control is in grey). Expression of CD16 and CD14 is shown in the ungated population for completeness in the side panels *A1* and *B1*, representing monocytes (black, CD14^+^), neutrophils (blue, CD14^−^CD16^high^), and lymphocytes (green, CD14^−^CD16^low^, likely NK cells). Since significant differences between groups were observed in the prevalence and cell number of CD14^high^CD16^−^ (black) and CD14^high^CD16^+^ (red) subsets, the level of CD16 expression was also specifically analysed within the CD14^high^ population, using an nMFI strategy. The histograms (*C* and *D*) show overlay of the baseline (empty histogram) and post-vaccination (filled histogram) intensity of fluorescence for CD16 of the stained samples (red). Also shown are the fluorescence levels of the antibody isotype controls (in grey, with dotted histogram and filled histogram for baseline and post-vaccination samples, respectively). The median fluorescence intensity (MFI) calculated in CD14^high^ monocytes at baseline (pre) and post-vaccination (post) was normalized by the MFI of the corresponding isotype control as indicated in the figure.

Monocyte characterization was performed accordingly with previously published methods.^9^ Briefly, 100 µL whole blood collected in ethylenediaminetetraacetic acid (EDTA) vacutainer tubes was incubated with saturating concentrations of both *R*-phycoerythrin (PE)-mouse anti-human CD14 (M5E2 clone; BD Bioscience, UK) and fluorescein isothiocyanate (FITC)-conjugated mouse anti-human CD16 (3G8 clone; BD Bioscience) for 20 min at 4°C. Isotype antibodies were used as negative control. Following erythrocyte lysis in BD FACS lysing solution (BD Bioscience), samples were washed in phosphate buffered saline containing 0.2% bovine serum albumin and 0.1% sodium azide, and then fixed in 1% paraformaldehyde. Immunostaining took place within an hour of the samples being obtained and data were acquired using a BD FACSCalibur (BD Bioscience) flow cytometer. A total of 100 000 events were acquired and post-acquisition analysis was performed using FlowJo (version 10) software (Tree Star, Ashland, OR, USA). Monocytes were identified on a forward (FSC) vs. side scatter (SSC) plot and gated to analyse expression of CD14 and CD16 fluorescence, in order to distinguish the different subsets of monocytes, including ‘classical’ CD14^high^CD16^−^, ‘intermediate’ CD14^high^CD16^+^ and ‘non-classical’ CD14^low^CD16^+^ cells. Percentage of each monocyte subset over total monocytes was calculated and absolute numbers were obtained based on full blood count. Every analysis was performed independently by two blinded researchers. In a subgroup of 10 participants, repeated measures of monocyte subset cell count were carried out on two consecutive measurements 2 days apart and before vaccine administration, in order to assess variability in monocyte subset cell count in the absence of any intervention (vaccination either in the presence or absence of pharmacological treatment). Such an analysis showed inter-assay variation of <2% for all the different monocytic subsets. Moreover, our gating strategy for monocyte characterization (which was based on a FSC vs. SSC monocyte profile) was directly compared with a flow cytometry methodology that also includes the pan-monocytic marker antibody, namely allopuricine-mouse anti-human CD86, in the antibody panel.^15^ In this case, cells with a typical monocytic FSC vs. SSC profile and positive to CD86 staining were gated to analyse CD14 and CD16 expression. Prevalence of the different monocyte subpopulations obtained with the two gating strategies was analysed on SPSS (version 23) to calculate the intraclass correlation coefficient (ICC) using an absolute agreement definition. The ICC was 0.961 (95% CI: 0.859–0.989; *P* < 0.0001), 0.924 (95% CI: 0.711–0.980; *P* < 0.0001), and 0.915 (95% CI: 0.695–0.977; *P* < 0.0001) for the classical, intermediate, and non-classical subsets, respectively (Supplementary material online, *Figure S1*). Moreover, in order to increase robustness of data in the analysis of changes in the phenotype of circulating monocytes 48 h post-immunization in the different groups, we also analysed the level of CD16 expression on monocytes using a normalized median fluorescence intensity (nMFI) strategy, in agreement with previously published methods used to monitor changes in cell immunophenotype in repeated measures.^16^ A representative analysis of flow cytometry data is shown in *Figure 1*.

### 2.5 Enzyme-linked immunosorbent assays and luminex assay

Enzyme-linked immunosorbent assays (ELISAs) were carried out to measure plasma levels of P-selectin (BEK1189, Biospes, China) using commercially available kits, as per manufacturer instructions. Inter-assay variability was found to be <5% for all the ELISA tests.

A custom-made magnetic Luminex^®^ ultrasensitive cytokine multiplex assay (Life Technologies Corporation, UK) was performed to measure levels of interleukin 1-β (IL-1β), interleukin-6 (IL-6), and tumour necrosis factor-alpha (TNF-α) in serum. Manufacturer instructions were followed and plates were analysed using a Luminex Flex Map 3D® analyser.

### 2.6 Sample size calculation

The sample size population for the study was calculated in accordance with previously published data that showed a standard deviation for CD16^+^ monocytes within a healthy population of 5%, with a difference in means between pre- and post-immunization values of 7.5%.^9^ Assuming a minimum detectable difference in means between the anti-platelet therapy and untreated groups at the end of the study of 4%; at power 0.9, and significance level of 0.05, this yielded a sample size of 15 per group.

### 2.7 Statistical analysis

Statistical analyses were performed using SPSS (version 23) software. A Shapiro–Wilk normality test was run for all variables measured in the study. The effect of treatments on monocyte subtypes, inflammatory biomarkers (high-sensitivity C-reactive protein and cytokines), and platelet activation (soluble P-selectin), which were all found not to be normally distributed, were compared using a non-parametric analysis of co-variance (rank ANCOVA) with Bonferroni *post hoc* correction for multiple comparison analysis, using baseline values as a covariate. Baseline vs. post-vaccination comparison of the analysed variables was also analysed within each group by Wilcoxon matched-pairs signed-rank test. Associations between variables were assessed by a Spearman's correlation analysis. A *P*-value of <0.05 was accepted as statistically significant. Data are expressed as the mean ± standard error of mean (SEM) or median and interquartile ranges (IQR) for parametric or non-parametric variables, respectively.

## 3. Results

### 3.1 Anti-platelet drugs do not influence high-sensitivity C-reactive protein rise post-immunization

**Figure 2:**
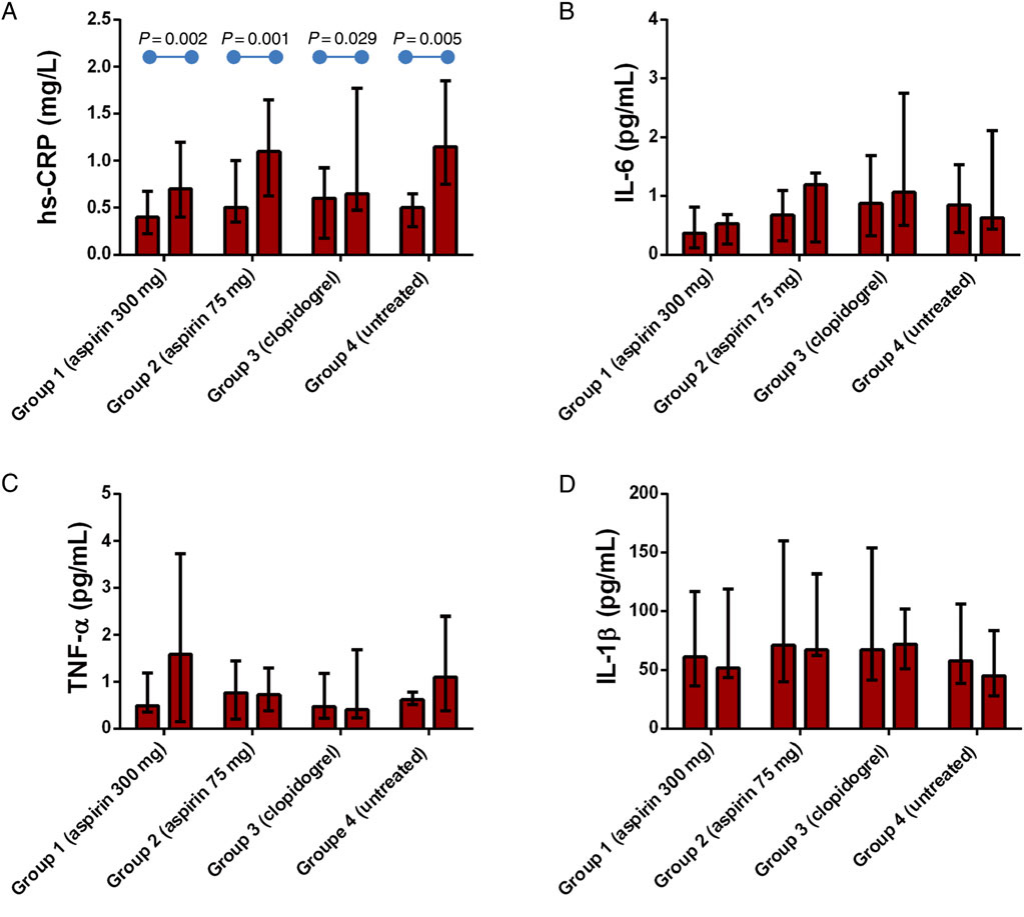
Anti-platelet drugs do not affect high-sensitivity C-reactive protein rise post-vaccination. The absolute values for high-sensitivity C-reactive protein (*A*), IL-6 (*B*), IL-1β (*C*), and TNF-α (*D*) at baseline and post-vaccination are shown. Data are reported as median with IQR. *P*-values were calculated using Wilcoxon matched-pairs signed-rank test for post-vaccination vs. baseline comparison within groups; rank ANCOVA was used for between-group comparison and identified no significant differences.

General characteristics of the study population stratified across groups are shown in *Table 1*. The influenza immunization induced an acute inflammatory response as evidenced by an increase in baseline high-sensitivity C-reactive protein post-vaccination in all groups (*Figure 2*). None of the anti-platelet drugs modified the rise in high-sensitivity C-reactive protein in response to vaccine administration (*Figure 2*).

**Table 1:** Baseline characteristics of the study population

	Group 1 (aspirin 300 mg)	Group 2 (aspirin 75 mg)	Group 3 (clopidogrel)	Group 4 (placebo)
Gender (*n*)	4 male	3 male	5 male	6 male
	11 female	12 female	10 female	9 female
Age (years)	32.00 (30.00–35.25)	33.50 (28.75–41.25)	36.00 (31.5–43.50)	37.00 (31.00–43.50)
SBP/DBP (mmHg)	117 ± 4/78 ± 2	121 ± 5/82 ± 3	123 ± 4/84 ± 2	125 ± 6/81 ± 4
Smokers (current) (*n*)	2	3	2	1
BMI (kg/m^2^)	22.78 (20.81–26.00)	25.23 (21.87–28.47)	25.12 (20.61–28.42)	23.63 (21.83–28.66)
HDL cholesterol (mmol/L)	1.872 ± 0.08	1.751 ± 0.10	1.735 ± 0.09	1.793 ± 0.09
LDL cholesterol (mmol/L)	2.74 (2.42–3.33)	2.87 (2.43–3.43)	2.49 (2.24–3.15)	2.20 (1.77–3.03)
Triglycerides (mmol/L)	1.08 (0.72–1.32)	1.00 (0.80–1.39)	1.07 (0.81–1.50)	0.93 (0.69–2.04)
Leucocytes (cells × 10^9^/L)	5.99 ± 0.31	6.01 ± 0.33	6.13 ± 0.38	6.23 ± 0.27
Monocytes (cells × 10^9^/L)	0.5 (0.4–0.5)	0.5 (0.4–0.5)	0.4 (0.3–0.5)	0.5 (0.4–0.625)
Platelets (×10^9^/L)	219.5 (190.3–240.8)	211.0 (184.0–240.0)	228.0 (204.0–251.0)	219.5 (171.3–248.8)
HbA1c (mmol/mol)	32.50 (30.75–36.00)	32.00 (29.75–34.00)	33.00 (31.00–34.00)	34 (31.00–36.00)
Creatinine (mmol/L)	73.53 ± 2.72	70.07 ± 3.27	72.79 ± 3.17	73.10 ± 3.54

Values are expressed as the mean ± SEM or median (IQR).

SBP, systolic blood pressure; DBP, diastolic blood pressure; BMI, body mass index; HDL, high-density lipoprotein; LDL, low-density lipoprotein.

There were no significant differences between post-vaccination and baseline levels of pro-inflammatory cytokines in any of the groups (*Figure 2*).

### 3.2 Expansion in the pool of intermediate CD14^high^CD16^+^ monocytes in response to influenza immunization is attenuated by anti-platelet treatment

**Figure 3:**
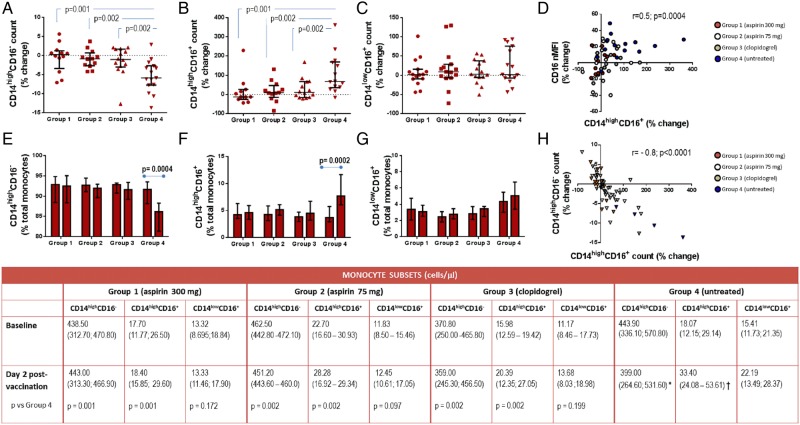
Anti-platelet drugs counteract CD14^high^CD16^+^ expansion induced by vaccination. The percentage change from baseline of CD14^high^CD16^−^, CD14^high^CD16^+^, and CD14^low^CD16^+^ cell count in each of the groups is reported in *A*, *B*, and *C*, respectively, with the absolute values at baseline and post-vaccination shown in the table (*P*-values were calculated using rank ANCOVA). Also shown is the prevalence of each monocyte subset (expressed as percentage over total monocytes) at baseline and post-vaccination in the different groups (*E*, *F*, and *G*). Graphs *D* and *H* show the correlation between percentage change in CD14^high^CD16^+^ cell count and CD16 nMFI (on the top) and between CD14^high^CD16^+^ and CD14^high^CD16^−^ cell count (on the bottom). Values are reported as median and IQR. *P = 0.002; **P = 0.0002 vs. baseline within Group 4 using Wilcoxon matched-pairs signed-rank test.

The baseline monocyte phenotype was similar among groups (*Figure 3*), with a typical preponderance of the classical CD14^high^CD16^−^ subtype (median of 92.7% of total monocytes) over the intermediate CD14^high^CD16^+^ (4.2%) and non-classical CD14^low^CD16^+^ (3.2%) subpopulations in all participants (*Figure 3*). While the total monocyte cell count remained stable post-vaccination in the whole study population, differences emerged among groups on the distribution of the different subsets. Indeed, the untreated participants showed a significant increase in both prevalence (from a median of 3.7 to 7.8% in median; *P* = 0.0002) and absolute number (from a median of 18.07 to 33.40 cells/µL; *P* = 0.0002) of CD14^high^CD16^+^ subtype; a concomitant reduction in the proportion (from a median of 91 to 86%; *P* = 0004) and cell count (from a median of 443.90 to 399.00 cells/µL; *P* = 0.002) of classical CD14^high^CD16^−^ monocytes was noted; no change in the non-classical CD14^low^CD16^+^ population was observed (*Figure 3*).

Treatment with all of the tested anti-platelet regimes counteracted the changes in monocyte phenotype induced by vaccination. Although no statistically significant differences were observed among the treated groups, aspirin 300 mg exerted the strongest effect in limiting the expansion of CD14^high^CD16^+^ count post-vaccination (median percentage increase from baseline was −12.6 in Group 1 vs. +67.3 in untreated; *P* = 0.001). Attenuation in the CD14^high^CD16^+^ increase post-vaccination was also observed in Group 2 (aspirin 75 mg) (median percentage increase was 9.3; *P* = 0.002 vs. untreated) and, although less pronounced, in Group 3 (clopidogrel) (median percentage increase was 10.8; *P* = 0.002 vs. untreated).

The results were confirmed when CD16 expression, specifically analysed within the CD14^high^ population, was studied using the nMFI strategy. Group 4 (untreated) showed a significant rise in monocytic CD16 levels post-vaccination (median percentage change from baseline was +20.92; *P* = 0.002 vs. baseline) that was abolished by all anti-platelet drugs, with aspirin 300 mg being the most efficacious among treatments (median percentage increase from baseline in Group 1 was −2.1; *P* = 0.006 vs. Group 4; in Groups 2 and 3, it was +0.5 and +2, respectively; *P* = 0.02 vs. untreated for both comparison). Likewise, a strong direct correlation was found between the increase from baseline of CD14^high^CD16^+^ cell count and CD16 nMFI (*r* = 0.5; *P* = 0.0004) (*Figure 3*). While attenuating the increase in CD14^high^CD16^+^ cells, the anti-platelet regimes counteracted the reduction in CD14^high^CD16^−^ monocytes observed in untreated participants post-vaccination (*Figure 3*). The two monocyte subsets showed a strong inverse correlation with one another (*r* = −0.8; *P* < 0.0001).

### 3.3 Modulation of P-selectin is linearly related to the change in monocyte phenotype

**Figure 4:**
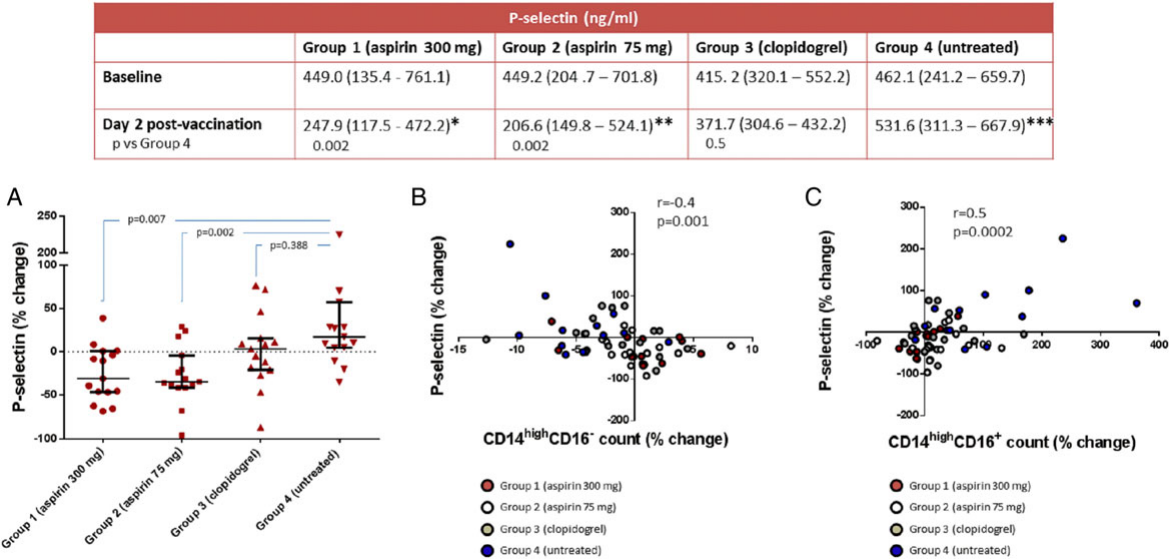
Modulation of P-selectin correlates with change in monocyte phenotype. The table shows the P-selectin values at baseline and post-vaccination stratified among groups, with *P*-values calculated using rank ANCOVA. Percentage change from baseline of P-selectin in each group is shown in graph *A. B* and *C* report the correlation between change in P-selectin and change in classical CD14^high^CD16^−^ and intermediate CD14^high^CD16^+^ monocyte cell count, respectively. Values are reported as median and IQR. *P = 0.003; **P = 0.011; ***P = 0.039 vs. baseline within groups using Wilcoxon matched-pairs signed-rank test.

The level of P-selectin post-immunization in Group 4 increased by a median of +17.2% (*P* = 0.039 vs. baseline) (*Figure 4*). A significant decrease in P-selectin was observed in participants on aspirin 300 mg (median percentage change from baseline was −30.7% in Group 1; *P* = 0.003 vs. baseline and *P* = 0.007 vs. untreated) and aspirin 75 mg (−34.7% in Group 2; *P* = 0.011 vs. baseline and *P* = 0.002 vs. untreated). Treatment with clopidogrel led to a median change from baseline of P-selectin of 3.3% (*P* = 0.463 vs. baseline and *P* = 0.388 vs. untreated). In a combined Spearman's correlation analysis incorporating all study variables, monocyte phenotype changes correlated only with changes in P-selectin. Indeed, the increase in P-selectin values was directly correlated with an increase in CD14^high^CD16^+^ cell count (*r* = 0.5; *P* = 0.0002) and a reduction in CD14^high^CD16^−^ absolute number (*r* = −0.4; *P* = 0.001) (*Figure 4*). No relationships emerged between P-selectin and any of the other variables analysed in the study population.

## 4. Discussion

Atheroma development in humans is accompanied by systemic immunological abnormalities that share homologies with autoimmune diseases.^17^ In a pathophysiological model of atherosclerosis, the key contributing factors can be grouped into three main phases: (i) accumulation within the artery and in blood of immunogenic self-proteins, i.e. oxidized low-density lipoprotein derived from exposure to cardiovascular risk factors; (ii) consequent activation of innate immune mediators (primarily myeloid cells, but recent findings also point towards platelets playing a crucial role) through engagement of pattern recognition receptors; and (iii) a subsequent adaptive immunological response involving T and B cells.^18^ In this context, the influenza immunization provides a valuable experimental model to study the dynamics of monocyte phenotype in response to an immunogenic stimulus. In keeping with a previous study by this group,^9^ the current data confirm that immunization administration generates a reduction in the proportion of “classical” CD14^high^CD16^−^ monocytes, which mirrors the increase in the ‘intermediate’ CD14^high^CD16^+^ subset in peripheral blood samples from the untreated participants. This shift in the profile of circulating monocytes towards a CD16^+^ phenotype mimics the inflammatory response that characterizes atherosclerotic patients.^2–8^ Whether this event has functional implications in disease progression remains unclear. It is worth mentioning, however, that CD16 is an important signalling molecule with a relevant role in the innate response to immunogenic stimuli, in light of its demonstrated involvement in the phagocytosis of immunoglobulin type G (IgG)-opsonized external particles by myeloid cell types that subsequently stimulates the adaptive immune system.^19,20^ Of note, polymorphism of CD16A, which is specifically expressed on monocytes, has been reported to influence susceptibility to, as well as severity of, coronary disease.^21^ Indeed, CD16A genetic variants confer distinct affinity for IgG molecules with consequent effects on the efficiency of clearance of circulating immunogenic/pro-atherogenic factors that would otherwise accumulate within the arterial wall to perpetuate inflammation.^21^ On this background, an increase in circulating CD14^high^CD16^+^ monocytes should be regarded as a protective anti-atherogenic mechanism. On the other hand, ‘intermediate’ monocytes are known to more easily infiltrate the arterial wall compared with the ‘classical’ CD14^high^CD16^−^ cells,^9^ thus suggesting a detrimental effect. However, in order to test the pro-atherogenic relevance of ‘intermediate’ monocytes in human disease, interventional clinical trials in cardiovascular patients would need to be conducted to establish whether therapeutic modulation of circulating monocyte profile impacts on plaque progression. To our knowledge, no such studies have been conducted to date. Our findings are the first to show a therapeutic modulation of the phenotype of circulating monocytes induced by therapies conventionally used in primary and secondary prevention of cardiovascular disease, whose potential effect on plaque progression would therefore merit consideration in future clinical trials.

In our study, all the tested anti-platelet regimes, including the COX-inhibitor, aspirin, used at high (300 mg) and low (75 mg) doses, and the P2Y12 antagonist clopidogrel were able to attenuate the expansion of ‘intermediate’ monocytes in the peripheral blood following vaccination. However, a different efficacy among treatments was noted in the modulation of the phenotype of circulating monocytes that was found to be linearly correlated with their effectiveness in reducing soluble P-selectin.

We have previously shown that expression of P-selectin on activated platelets and the consequent formation of monocyte–platelet aggregates (MPA) is a key event in the acquisition of a CD16^+^ profile by circulating monocytes.^9^ In the current study, we did not perform MPA measurement due to technical limitations. Indeed, while the monocytic phenotype remains stable in blood collected in EDTA tubes for up to 4 h since venepuncture,^22^ immediate blood processing is required for a reliable assessment of MPA in citrated plasma that is not affected by *in vitro* platelet activation.^23^ In this study, plasma separation could be performed soon after blood sampling, while the staining for flow cytometric assays was delayed by ∼30 min following venepuncture. Hence, soluble P-selectin was chosen as a platelet marker to provide a more reliable evaluation of platelet activity and an indirect measure of monocyte–platelet interaction, particularly in consideration of our prior *in vitro* experiments that showed a significant rise in soluble P-selectin during MPA formation^9^ and recent clinical evidence describing a significant contribution of soluble P-selectin to MPA formation *in vivo*.^24^

The different effects observed among treatments on the level of P-selectin, particularly between the two aspirin groups and clopidogrel, might be attributed to either a different pharmacological target, i.e. COX-inhibition vs. P2Y12 antagonism, or different pharmacokinetics. Indeed, aspirin achieves a dose-dependent maximum platelet inhibition within 2 h post-loading dose while clopidogrel requires 5 h to reach a steady state and a level of platelet inhibition of 60% only following a loading dose of 300 mg.^25^ These differences might have played a critical role particularly in a short-term study such as this. As regards the two different groups of aspirin, the slight superiority of the 300 mg dose might be ascribed to the dose-dependent inhibition of COX-activity with a consequent anti-inflammatory action that for low doses of aspirin (75 mg) is negligible.^26^ However, aspirin 300 mg did not modify the inflammatory biomarkers measured in this study, consistent with early evidence demonstrating a clinically relevant anti-inflammatory effect of aspirin occurring at doses of 1 g or greater.^27^ Overall, this evidence points towards platelet inhibition as the main determinant in the observed results, which is particularly supported by the direct relationship found between amplitude in P-selectin reduction and change in monocyte phenotype in response to anti-platelet therapy. Similarity between the two groups of aspirin appears of particular relevance in light of the fact that aspirin 75 mg is the dose currently recommended for cardiovascular prevention, and no superior anti-thrombotic efficacy has been reported for aspirin 300 mg.^28–33^

The lack of activity of anti-platelet therapy on the non-specific marker of inflammation, high-sensitivity C-reactive protein, suggests a targeted immunomodulatory action. Regarding production of pro-inflammatory cytokines, the phenotype of circulating monocytes did not influence the levels of TNF-α, IL-6, and IL-1β in our study participants, at least when measured 48 h post-immunization. There were no significant differences between their baseline and post-immunization values in any of the groups. This is consistent with previously published reports showing an increase in high-sensitivity C-reactive protein in healthy subjects 2 days after influenza immunization that can be partly ascribed to an early rise in IL-6, but not TNF-α, 1 day post-vaccination.^34,35^ We cannot exclude, therefore, a change in levels of inflammatory cytokines among groups at time points other than those considered in the current study. Also, the effect of treatment on monocyte distribution was not analysed beyond the 48 h post-immunization; thus, the total duration of this effect remains to be established.

In conclusion, we have demonstrated in this proof-of-concept study that anti-platelet therapy can attenuate the development of a CD16^+^ profile by circulating monocytes under pro-inflammatory conditions. Modulation of P-selectin levels seems to be critical in determining the extent of this pharmacological action on monocytes, possibly linked to an interference in MPA formation; however, this remains to be confirmed. Further work is needed to better understand the underlying biomechanistics. Indeed, a limitation of this study was the lack of inclusion of additional platelet biomarkers and functional assays. These may have highlighted modulatory actions of anti-platelet drugs on specific platelet-dependent pathways. The relationship found between P-selectin levels and monocyte phenotype could merely reflect effectiveness of platelet inhibition without this biomarker, necessarily representing a mediator of change in monocyte phenotype. More importantly, further research is needed to clarify whether a similar therapeutic effect is exerted by anti-platelet therapy in cardiovascular patients and to ascertain its implication in atherosclerosis progression.

## Supplementary material

Supplementary material is available at *Cardiovascular Research* online.

## Funding

This work was supported by a grant from the British Heart Foundation (ref. FS/13/45/30345). Funding to pay the Open Access publication charges for this article was provided by…
